# Methodological Considerations Regarding the Prognostic Utility of “Life's Essential 8” in Sleep Disorder Populations: A Letter to the Editor

**DOI:** 10.1002/clc.70356

**Published:** 2026-05-25

**Authors:** Fatima Masood Khan, Mohsin Zahur, Muhammad Usman Faiz

**Affiliations:** ^1^ Abbottabad International Medical College Abbottabad Pakistan; ^2^ Khyber Medical University Peshawar Pakistan

To the Editor,

We read with considerable interest the prospective cohort study by Wei et al., investigating the prognostic value of the Life's Essential 8 (LE8) score for all‐cause and cardiovascular disease (CVD) mortality among adults with sleep disorders using NHANES data (2005–2014). The well‐structured analysis incorporating weighted Cox proportional hazards models, restricted cubic spline analyses, and machine learning (ML) algorithms feels like a meaningful contribution toward cardiovascular health optimization in high‐risk populations. We respectfully wish to raise three methodological considerations that may further contextualize these findings (though, maybe, also help the reader interpret what is truly going on).

Sleep disorders encompass pathophysiologically distinct entities, including obstructive sleep apnea (OSA), insomnia, and restless legs syndrome, each exerting divergent cardiovascular effects [1]. OSA drives cardiovascular risk mainly through intermittent hypoxia and sympathetic hyperactivation, whereas insomnia seems to operate via hypothalamic pituitary adrenal axis dysregulation and sustained cortisol elevation. Javaheri et al. have broadly characterized these mechanistic differences and their disparate cardiovascular sequelae [2]. By combining all subtypes into a single “sleep disorder” category, because the subtype‐specific NHANES questionnaires were discontinued after 2007–2008, the study risks introducing clinical heterogeneity that could blunt or even misrepresent the true LE8 mortality relationship within each subtype. Future work using objective polysomnographic data should stratify by disorder subtype for more precise and practically useful findings. The authors do acknowledge this constraint in the limitations section, but we think its implications for the directionality and magnitude of the LE8 mortality estimates deserve stronger methodological emphasis than what is currently provided.

A second issue concerns the use of one baseline LE8 assessment across a median follow‐up of 103 months. The LE8 framework includes inherently dynamic parameters, that is, dietary habits, physical activity, BMI, blood glucose, blood pressure, and lipid levels, each of which may shift a lot fairly over nearly a decade, especially under evolving pharmacological management. The original LE8 advisory by Lloyd‐Jones et al. explicitly highlights the longitudinal nature of cardiovascular health metrics, framing CVH status as a dynamic continuum instead of a fixed trait [3]. A single baseline snapshot cannot fully capture participants' cumulative cardiovascular health trajectories, and this may generate non‐differential misclassification bias that would, in turn, attenuate the estimated effect sizes. Using time updated LE8 measurements, even on a biennial rhythm consistent with subsequent NHANES survey cycles, would better describe the longitudinal exposure outcome relationship, and also support stronger causal inference.

Third, the clinical generalizability of the proposed ML predictive models is meaningfully limited by the lack of external validation. Although rigorous fivefold cross‐validation was applied within the training cohort, the models were essentially developed and assessed only within one historical NHANES data set. The TRIPOD (Transparent Reporting of a multivariable prediction model for Individual Prognosis or Diagnosis) statement, the methodological gold standard for clinical prediction model appraisal, explicitly requires external validation on independent cohorts as a non‐negotiable step before wider clinical adoption [4]. Moreover, prediction models built from a single national data set are known to show noticeable performance declines when applied to geographically and ethnically diverse populations [5]. Therefore, prospective validation across separate, independent, and diverse cohorts is essential to confirm the clinical dependability of the Gradient Boosting Decision Tree model before routine use. The authors note this limitation themselves, yet we argue the issue is particularly consequential for ML‐based models, which are generally more vulnerable to cohort‐specific overfitting than traditional Cox regression approaches.

In conclusion, we commend Wei et al. for this pioneering investigation, which appears to be the first prospective cohort study evaluating the prognostic significance of LE8 within a sleep disorder population. Taking up these methodological considerations in subsequent research would substantially improve the rigor, reproducibility, and real‐world clinical relevance of these important observations.

## Author Contributions

The authors have read and approved the final version of this letter.

## Funding

The authors have nothing to report.

## Ethics Statement

The authors have nothing to report.

## Consent

The authors have nothing to report.

## Conflicts of Interest

The authors declare no conflicts of interest.

## Data Availability

Data sharing is not applicable to this article as no data sets were generated or analyzed during the current study.
